# Optimization of alkaline protease production by rational deletion of sporulation related genes in *Bacillus licheniformis*

**DOI:** 10.1186/s12934-019-1174-1

**Published:** 2019-07-25

**Authors:** Cuixia Zhou, Huiying Zhou, Huitu Zhang, Fuping Lu

**Affiliations:** Key Laboratory of Industrial Fermentation Microbiology, Ministry of Education, College of Biotechnology, Tianjin University of Science & Technology, No. 29, 13th Road, Tianjin Economic-Technological Development Area, Tianjin 022, 300457 People’s Republic of China

**Keywords:** Sporulation, Asporogenic mutants, Alkaline protease, *Bacillus licheniformis*

## Abstract

**Background:**

Our laboratory has constructed a *Bacillus licheniformis* strain that secretes alkaline protease (AprE) with excellent enzymatic properties. *B. licheniformis* is generally regarded as safe and has a high industrial exoenzyme secretion capacity, but the host retains some undomesticated characteristic that increase its competitiveness and survival, such as spore-formation, which increases the requirements and difficulties in industrial operations (e.g. sterilization and enzyme activity control). Furthermore, the influence of sporulation on alkaline protease production in *B. licheniformis* has not been elucidated in detail.

**Result:**

A series of asporogenic variants of the parent strain were constructed by individually knocking out the master regulator genes (*spo*0A, *sig*F and *sig*E) involved in sporulation. Most of the variants formed abortively disporic cells characterized by asymmetric septa at the poles and unable to survive incubation at 75 °C for 10 min. Two of them (Δ*sig*F and Δ*sig*E) exhibited superior characteristics in protease production, especially improving the expression of the *apr*E gene. Under the currently used fermentation conditions, the vegetative production phase of Δ*sig*F can be prolonged to 72 h, and the highest protease production of Δ*sig*F reached 29,494 ± 1053 U/mL, which was about 19.7% higher than that of the wild-type strain.

**Conclusion:**

We first constructed three key sporulation-deficient strain to investigate the effect of sporulation on alkaline protease synthesis. The *sig*F mutant retained important industrial properties such as facilitating the sterilization process, a prolonged stable phase of enzyme production and slower decreasing trend, which will be superior in energy conservation, simpler operations and target product controlling effect. In summary, the work provides a useful industrial host with preferable characteristics and a novel strategy to enhance the production of protease.

**Electronic supplementary material:**

The online version of this article (10.1186/s12934-019-1174-1) contains supplementary material, which is available to authorized users.

## Introduction

Alkaline protease is an industrially important hydrolytic enzyme that acts on the peptide bonds within the structure of proteins and is active even in the presence of organic solvents [1]. Consequently, it occupies the largest part of the global protease market share [2]. Since alkaline protease possesses excellent features of high catalytic power, specificity, water solubility, non-toxicity, edibility, environmental friendliness, etc. [3], it has been used in a wide-range of applications in the fields of detergents, leather processing, waste management, as well as the food and pharmaceutical industries [4].

In general, *Bacillus* species are efficient alkaline protease producers [5, 6], and especially *Bacillus licheniformis* is a promising industrial host strain for protein production, partly due to its ‘Generally Recognized as Safe (GRAS)’ status and its high enzyme secretion capacity [7, 8]. *B. licheniformis* is widely distributed in soils where it helps recycle nutrients by producing and secreting macromolecule-degrading hydrolases such as amylases, proteases, cellulases and phosphatases [9]. In the challenging environment with a discontinuous supply of nutrients, abiotic stresses and competition from cohabiting microbes, *Bacillus* strains have developed a series of strategies to increase their competitiveness and survival, spore-formation being perhaps the best-known [10–12]. However, in industrial fermentation processes the microorganism is often confronted with similar adverse conditions (oxidative, osmotic stress, starvation for nutrients), and the bacteria can form spores to resist the hostile physical and chemical impacts in the bioreactor [13], which increases the requirements and difficulties of industrial operations such as incomplete sterilization, and reduced enzyme yield [14]. Most fermentation processes are susceptible to microbial contamination and require an energy-intensive sterilization process, which increases energy consumption and process complexity, contributing to the high costs of bio-products [15]. Sporulation is a “last resort” response to nutrient deprivation and stress [16] that includes developmental changes in cellular morphology, biochemistry and physiology. Due to its unique biological aspects, it has attracted interest from bacteriologists, developmental biologists and those interested in the practical aspects of spore formation [17].

The complex regulatory network of sporulation and the relationship between the involved factors have been largely elucidated in recent years [18]. On this basis, many efforts have been made in the development of industrial to improve the production of target proteins or inhibit the synthesis of toxic substances by escaping from detrimental sporulation. For example, deletion of *spo*0A in *Clostridium tyrobutyricum* led to high-level butanol production [19], while insertional inactivation of *spo0A* in the *Clostridium botulinum* type E strain resulted in significantly reduced production of botulinum neurotoxin [20]. Also, disruption of *spo*IIAC (*sig*F) for greater resistance to spore formation and increased secretion of β-cyclodextrin glycosyltransferase into the extracellular medium was reported in *Bacillus subtilis* [21], and one study found the greatest effect on the enhancement of enzyme productivity in the *sig*E-deleted mutant of *B. subtilis* [22]. While it had been reported that the production of alkaline protease is associated with the onset of sporulation in *B. subtilis* [23], the expression of eight genes encoding extracellular proteases was very poor or absent in the *spo*0A mutant [24]. Interestingly, almost all reports on the initiation of sporulation in the cases of *B. subtilis* found an association with such phenomena as protease production [25, 26]. There are some interspecific differences in physiological and other metabolic mechanisms between *B. subtilis* and *B. licheniformis*, although the phylogenetic relationship of the two species is close [27]. However, few studies to date have systematically investigated the effects of sporulation on the production of alkaline protease in *B. licheniformis*. To our best knowledge, the sole exception is a single report on the production of extracellular enzymes in a *spo*IIAC mutant of *B. licheniformis* [28]. Accordingly, it is necessary and valuable to investigate the effect of sporulation on the production of alkaline protease and construct a highly productive chassis for industrial applications based on *B. licheniformis* cells without the undomesticated sporulation-related properties.

Consequently, we explored the production of alkaline protease in different sporulation-deficient strains of *B. licheniformis*. Here, we constructed three asporogenic mutants by disrupting *spo*0A, an initial response-regulator protein for entry into sporulation, and the two cell-specific sigma factors σ^F^ and σ^E^, which are activated shortly after asymmetric division and direct gene expression in the mother cell and forespore, respectively. The stability, viability, cell lysis, enzyme productivity and other relevant properties of the mutants were systematically investigated in order to inspect the effect of sporulation on cell growth and production of alkaline protease and to establish a stable food-grade *B. licheniformis* system as an industrial workhorse for the production of extracellular enzymes. Taken together, this work broadens our understanding of the relationship between sporulation and extracellular enzyme synthesis and provides a practical method to eliminate or avoid the undomesticated properties of *Bacillus*-based cell factories.

## Materials and methods

### Bacterial strains and culture conditions

All the strains used in this research are listed in Table 1. *B. licheniformis* 2709, BL Δ*upp* was used as the original strain for genetic modifications, and the *E. coli* strains EC135 and EC135 pM.Bam were used for plasmid construction and methylation, respectively [29]. The shuttle expression plasmid pWH1520 was used to construct the gene knockout vectors.

**Table 1:** Strains and plasmids used in the study

Strain or plasmid	Characteristics or purpose	Reference
Strains		
*E. coli* EC135	Knockout vectors construction	Chinese Academy of Science
*E. coli* EC135 pM.Bam	Plasmid DNA methylation modification	Chinese Academy of Science
*B. licheniformis* 2709 Δupp (BL Δupp)	Parent host	This work
*B. licheniformis* ΔsigF (BL ΔF)	Δ*sigF*, *sigF* gene deletion	This work
*B. licheniformis* ΔsigE (BL ΔE)	Δ*sigE*, *sig E* gene deletion	This work
*B. licheniformis* Δspo0A (BL Δ0A)	Δspo0A, *spo0A* gene deletion	This work
*B. licheniformis* CsigF (BL CF)	*sigF* gene complementation	This work
*B. licheniformis* CsigE (BL CE)	*sigE* gene complementation	This work
*B. licheniformis* Cspo0A (BL C0A)	*spo*0A gene complementation	This work
Plasmids		
pWH1520	Shuttle expression vector, Amp^r^ (*E. coli*) and Tet^r^ (*Bacillus*): MCS	Nankai University
pWHU	pWH1520, *upr* T gene, cas9 gene	This work
pWHF	Knockout vector, *spo* IIAC gene deletion	This work
pWHE	Knockout vector, *sig E* gene deletion	This work
pWHA	Knockout vector, *spo0A* gene deletion	This work
pWHCF	Backcrossed vector, spo IIAC gene complementation	This work
pWHCE	Backcrossed vector, *sig E* gene complementation	This work
pWHCA	Backcrossed vector, *spo0A* gene complementation	This work

LB medium was used as the basic medium for bacterial growth of *Bacillus* and *E. coli*, and the corresponding titers of antibiotics (100 μg/mL ampicillin, 50 μg/mL spectinomycin, 20 μg/mL tetracycline, 30 μg/mL 5-fluorouracil) were added to the medium if necessary. The seed cultures (50 mL LB medium) were cultivated in a 250 mL flask at 220 rpm and 37 °C until the OD_600_ reached 1.2. The medium for production of alkaline protease was composed of 64 g/L corn starch, 40 g/L soybean meal5, 4 g/L Na_2_HPO_4_, 0.3 g/L KH_2_PO_4_, 0.7 g/L thermostable amylase (Biotopped, Beijing, China), pH 7.2. The inoculum (2%) was added into 100 mL of fermentation medium in a 500 mL flask, and was incubated at 220 rpm and 37 °C for the indicated time. All the fermentation experiments were performed at least three times.

### Gene knockout and genetic complementation in *B. licheniformis* 2709 BL Δ*upp*

**Fig. 1:**
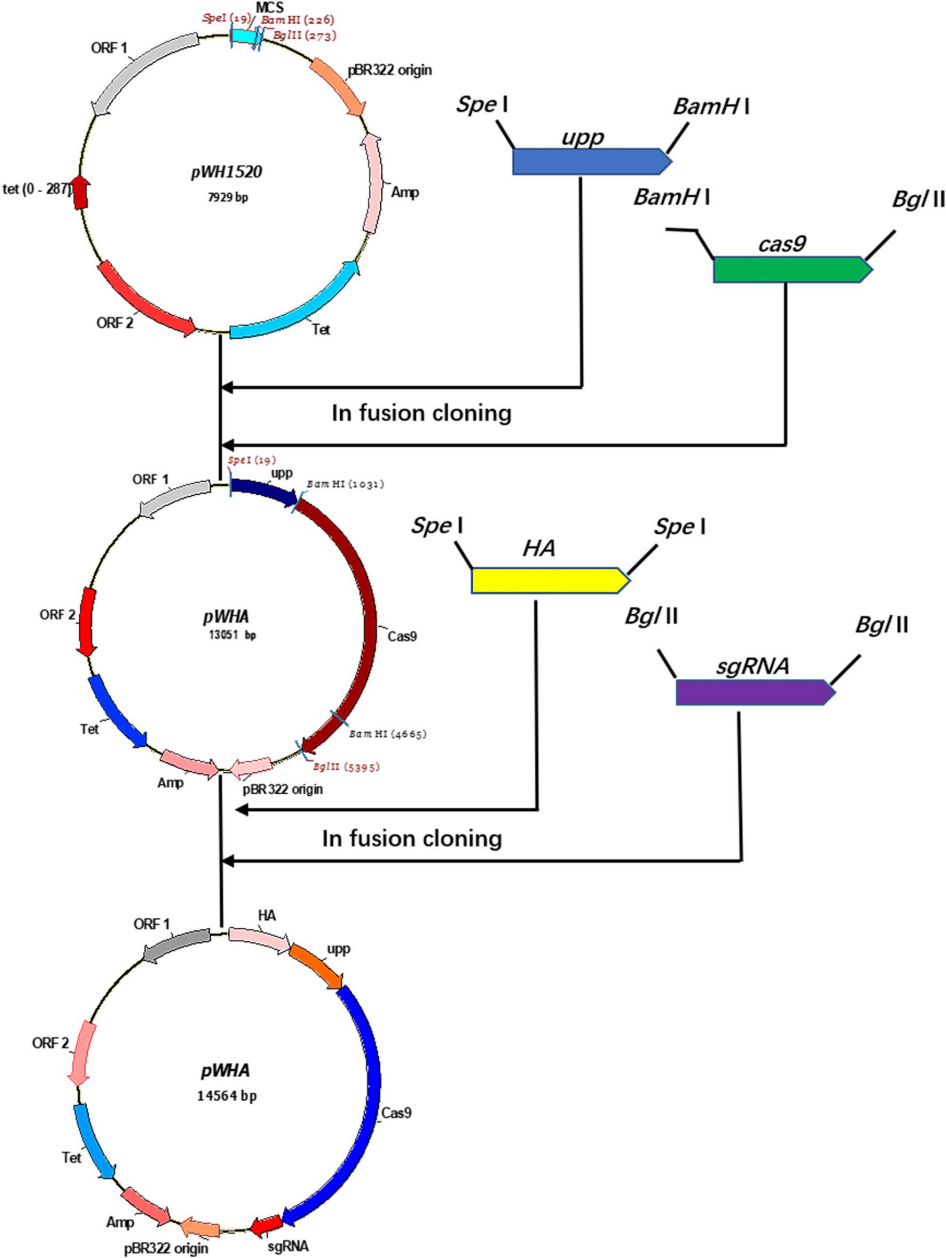
The construction procedure of the knockout plasmid pWHA

The genes *spo*0A, *spo*IIAC and *sig*E were deleted individually in BL Δ*upp* according to a previously reported method [7], as follows: First, the homologous arm (HA) was integrated into the vector: the left arm (L) and right arm (R) regions of *spo*0A were respectively amplified using primer pairs 0A-LF/0A-LR and 0A-RF/0A-RR (Additional file 1: Table S1), and fused by splicing overlap extension (SOE)-PCR using the primer pair 0A-LF/0A-RR. The fused fragment was cloned into the pWHU vector at the *Spe*I restriction site. Subsequently, the synthesized sgRNA transcription cassette was integrated into the knockout vector, yielding pWHA, which was confirmed by diagnostic PCR and DNA sequencing (Fig. 1).

Then, the recombinant plasmid was transferred into BL Δ*upp* by electrotransformation and tetracycline-resistant transformants were verified by colony PCR and plasmid extraction. The positive transformants were streaked onto LB plates and grown at 37 °C for about 12 h, after which diagnostic PCR (A-VF/A-VR) was used to verify the mutants, and DNA sequencing confirmed that the *spo0A* deficient strain (BL *ΔA*) was constructed successfully. The *spoIIAC* and *sigE* deficient strains (BL *ΔF* and BL *ΔE*) were constructed in analogy to the construction of BL *ΔA*. The complemented strains were respectively constructed by introducing each of the specific backcrossed vectors including the complementary gene (Table 1) into the mutants using the same gene editing method. The main primers used for gene complementation were listed in the additional supporting file (Additional file 1: Table S2).

### Characterization of the mutants

#### Morphological characteristics

To determine the effects of sporulation on the cellular morphology of the bacteria, the morphological changes of different strains in the study were investigated via light microscopy (LM) (Nikon ECLIPSE Ci, made by Nikon corporation, Tokyo, Japan) to observe cell morphology by Crystal Violet stain and to visualize spores by Schaeffer–Fulton stain, and further confirmed in more detail via scanning electron microscopy (SEM). SEM was carried out based on reported method [30] with minor modifications as follows: Cells grown in LB medium or fermentation medium were collected by centrifugation (5000×*g* for 5 min) and washed in phosphate buffered saline (PBS) three times. Samples were fixed using 2.5% (v/v) glutaraldehyde in PBS overnight and washed three times with PBS to remove the remaining glutaraldehyde, then covered with platinum using a Q150R rotary-pumped sputter coater before observation using an SU8010 FE-SEM (Hitachi, Japan).

#### Viability of the strains

In order to study the effect of sporulation on the strains’ viability, the optical density at 600 nm (OD_600_), cell lysis and viable count were investigated. The growth curves of the strains were obtained using a microplate reader. First, an independent colony was transferred into a 250 mL flask with 50 mL of fresh LB medium and shaken at 37 °C, then the OD_600_ of the bacterial suspension (200 μL/microwell) was detected using the Infinite 200PRO microplate reader (TECAN, Austria) every 2 h. Cell lysis was inspected by observing cell morphology under the LM, and counting the viable bacteria according to the method noted by the national standardization administration commission [31].

#### Sporulation efficiency

Sporulation efficiency of the strains in LB and fermentation medium after cultivation for different times was determined on an LB plate cultured for 12 h at 37 °C, as colony-forming units (cfu) per mL (spores) after heat treatment by incubation at 75 °C for 10 min, compared with cfu per ml (viable count) of the unheated treatment sample.

### Alkaline protease synthesis in different mutants

The alkaline protease productivity of the mutants and parent strain were studied using sampling at different incubation times in shake-flask fermentations. We investigated the alkaline protease activity in culture supernatants using the method according to the national standardization administration commission [32], observed the morphological changes by microscope and accurately counted the live bacteria of different samples; then alkaline protease activity per unit biomass were obtained. To evaluate the utility of the best engineered strain as a host for extracellular protein expression, the ability of the mutant possessing better alkaline protease production properties in shake flasks was compared with that of the parent strain in a 5-L fermenter.

### Analysis of transcription levels

The strains used in the study were cultured in liquid fermentation medium for 60 h at 37 °C and the cells were collected at different cultivation times corresponding to the exponential phase and stable phase of alkaline protease activity per unit biomass. Total RNA was extracted using TRIzol^®^ Reagent (Promega, USA) and the quality of the RNA was determined by agarose gel electrophoresis and the NanoDrop 1000 spectrophotometer (Thermo Scientific, USA). Trace DNA was digested using RNase-free DNase I (TaKaRa, Japan), and the first strand of cDNA was amplified using RevertAid First Strand cDNA Synthesis Kit (Thermo, USA). To investigated the expression levels of alkaline protease genes, quantitative real-time PCR (qRT-PCR) were performed in an ABI Stepone Real-Time PCR System (Stepone plus, Thermo Scientific, USA). The primers in Additional file 1: Table S1 were used for amplifying the alkaline protease gene (AP-F/AP-R) from the BL *Δupp* strain and the three other mutants, and 16S rRNA (S-F/S-R) served as the reference gene to normalize the data. The transcriptional levels of the alkaline protease gene in the mutants at different culture times were compared with those of the control strain BL *Δupp* after normalization to the reference gene 16S rRNA using the 2^−ΔΔCt^ method. All the experiments were repeated three times.

### Batch fermentation

Fed-batch fermentations were carried out with various strains in a 5-L bioreactor (MC-GS, Shanghai Bai Lun biological technology Co., Shanghai, China) with a 3-L working volume according to the optimum conditions of batch fermentation. The relevant fermentation media used in the study encompassed buttermilk plates comprising 4 g/L casein in LB; liquid LB medium as the first-degree seed medium; and second-degree seed medium comprising corn starch 50 g/L, soybean meal 30 g/L, Na_2_HPO_4_ 4 g/L, KH_2_PO_4_ 0.3 g/L, 0.5 g/L thermostable amylase, pH 7.3. The strain was first incubated in first-degree seed medium (50 mL/250 mL) at 37 °C for 8 h, then the bacterial suspension was streaked onto buttermilk plates and cultured at 37 °C for 36 h. The colony with relevant bigger transparent zone around it was picked into the first-degree seed medium (50 mL/250 mL) and grown at 37 °C for 8 h, and the resulting seed was transferred into the second-degree seed medium at 4% inoculum size and cultivated at 37 °C for 5–7 h. Finally, the active seed culture was used to inoculate the fermentation medium at a volume ratio of 5%. The fermentation process was carried out at pH 7.3 and 37 °C. The changes of other parameters as well as the production of alkaline protease along with the fermentation in 5-L fermenter were investigated. The dissolved oxygen and pH were self-tested by the fermenter; the biomass was indicated by viable count, and the reducing sugar was assayed by the DNS method.

### Statistical analyses

All experiments were conducted in triplicate, and the experimental data were expressed as the means ± standard deviations for each sample point. The significance of differences was assessed using two-way ANOVA (P < 0.05).

## Results

### Construction of sporulation-deficient strains

**Fig. 2:**
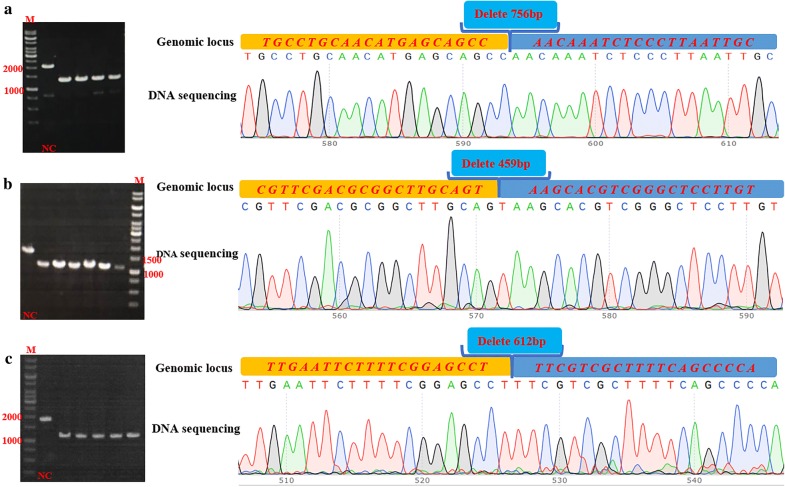
Confirmation of disruption of *spo*IIAC, *spo*0A and *sig*E. The DNA sequencing, alignment result of the deletion-carrying mutant and agarose gel electrophoresis; **a** *spo*IIAC deletion, losing 756 bp; **b** *spo*0A deletion, losing 459 bp; **c** *sig*E gene deletion, losing 612 bp. M: nucleic acid marker; NC: The negative control was obtained by colony PCR of the wild-strain

Based on the genome sequence and annotation of *B. licheniformis* 2709 in our lab, three genes (*spo*0A, *sig*E, *spo*IIAC) encoding spore formation factors were selected. In order to investigated the effect of sporulation on protease synthesis, the *spo*0A, *sig*E, *spo*IIAC genes were deleted in the parent strain *B. licheniformis* 2709 BL *Δupp*, respectively. In the case of the construction of the *spo*0A knockout plasmid pWHA as an example (Fig. 1), the bands amplified from the mutants had the approximate length of the HA, as well as the sequencing result of the PCR product from the mutant (Fig. 2), confirming that *spo*0A was disrupted precisely and successfully. The new mutant strain was named *B. licheniformis* BL *ΔA*. Similarly, the *sig*E and *spo*IIAC genes were deleted using the same method, and the resulting mutants were named *B. licheniformis* BL *ΔE* and *B. licheniformis* BL *ΔF*, respectively. To prove the observed phenotypes were due to the introduced mutations and to exclude side mutations that may be occasionally produced by CRISPR/Cas9 system, the backcrossed experiment was carried out. The complementary strains were respectively generated by genomic integrating genes of *spo*0A, *sig*E, *spo*IIAC, called BL C0A, BL CE and BL CF, which were verified by colony PCR shown in the appendix file (Additional file 1: Fig. S1). The phenotypes (colony form, color, cell growth and enzyme activity) were observed and the results showed no significant difference among the complementary strains and the wild-type strain (data not shown).

### Characterization of the mutants

**Fig. 3:**
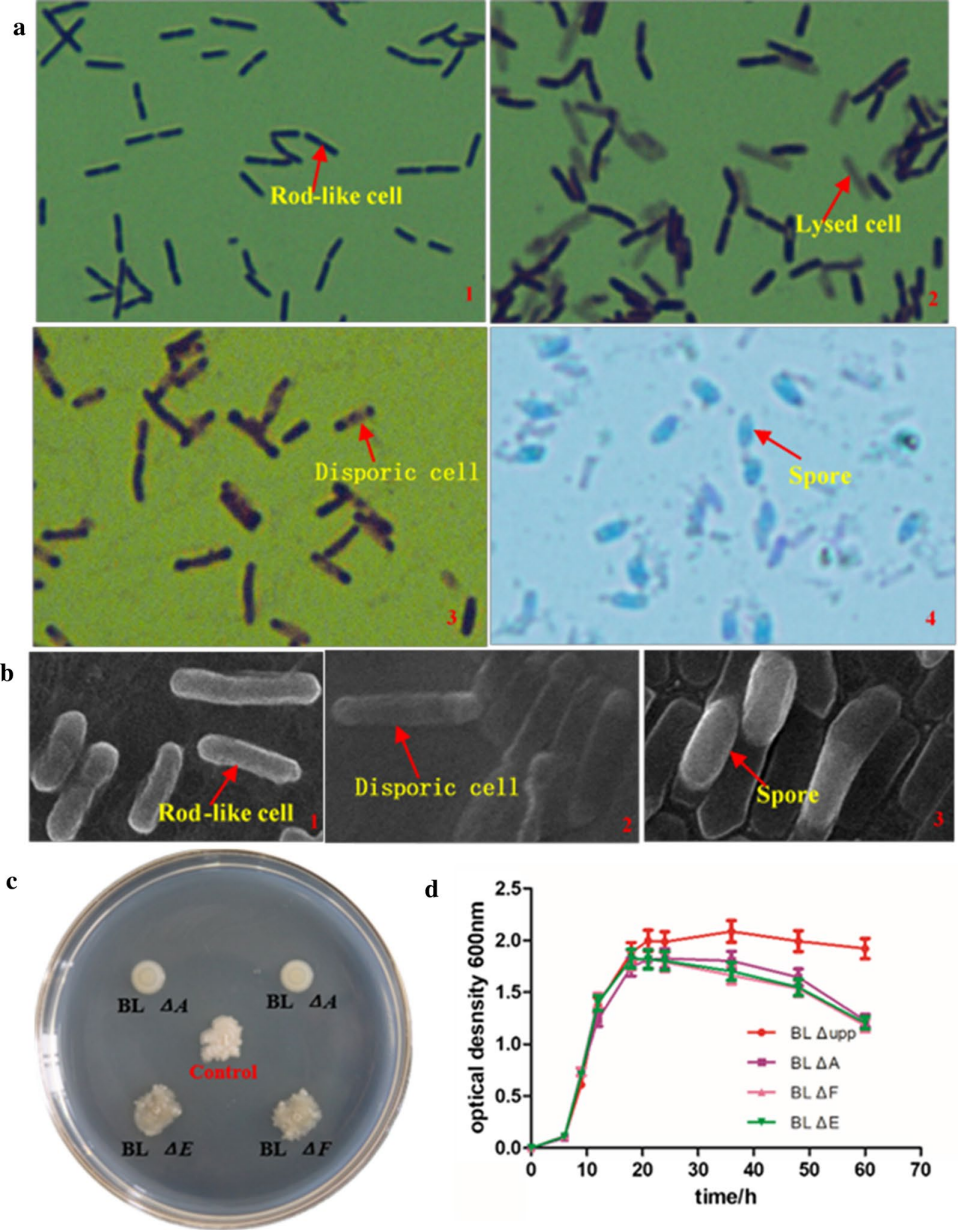
The characterization of the sporulation-deficient mutants and wild-type strain. The red arrow gave directions of every typical morphology (×100). **a** The cell morphology was observed by crystal violet (a1–a3) staining assay and Schaeffer-Fulton staining assay (a4): a1, “rod” like cellular morphology of the strains under the optical microscopy (OM); a2, cellular morphology with cell lysis of the *spo*0A mutant under OM; a3, an abortively disporic cell with two asymmetric septa under OM; a4, mature endospores in the parent strain under OM. **b** The cell morphology was observed under scanning electron microscopy (SEM) (×5000): b1, “rod” like cellular morphology of the strains under SEM; b2, an abortively disporic cell with two asymmetric septa under SEM; b3, mature spores in the parent strain under SEM. **c** The colonial morphology of the mutants and control strain in LB plate. **d** The cell growth curve of different strains

The deletion strains BL *ΔA*, BL *ΔE* and BL *ΔF* were used for further study. The morphological changes of the three mutations in LB medium at different times were observed using LM and SEM, and the effect of the mutations was clearly visible. When the incubation time was 12 h there was no difference in cell morphology (rod-like cells) among the mutants and the parent strains (Fig. 3a1, b1). However, when the incubation time was 24 h, BL *ΔE* and BL *ΔF* had similar cellular morphology with two asymmetric septa in an abortively disporic cell (Fig. 3a3, b2), but the BL *ΔA* still retained rod-shaped cells (Fig. 3a1, b1), and the endospores began to appear in the parent strain (Fig. 3a4) with a sporulation efficiency of 31.3% (Table 2). All the deletions gave rise to completely asporogenic strains, even after a time period in which the parent strain differentiated into mature spores with a sporulation efficiency of 100% (Fig. 3a4, b3). Cellular growth was further distinguished by observing cellular morphology under the electron microscope (Fig. 3b), which validated the results obtained by LM. Furthermore, the morphology of the strains’ colonies on LB plates was compared and there were obvious differences in form or color. The colony form of BL *ΔE* and BL *ΔF* was random, which was similar to that of the parent strain except for the colony color. By contrast, the colonies of BL *ΔA* were tidy and round (Fig. 3c).

**Table 2:** Viable cell count and sporulation efficiency of different strains in LB medium

Time/h	BL *Δupp*		BL *ΔF*		BL *ΔE*		BL *ΔA*	
	cfu/mL	spo rate/%	cfu/mL	spo rate/%	cfu/mL	spo rate/%	cfu/mL	spo rate/%
12	7.9 × 10^8^	0	7.7 × 10^8^	0	8.1 × 10^8^	0	2.4 × 10^9^	0
24	2.6 × 10^9^	31.3	2.5 × 10^9^	0	2.5 × 10^9^	0	4.2 × 10^9^	0
36	2.7 × 10^9^	88.2	2.4 × 10^9^	0	2.5 × 10^9^	0	2.3 × 10^9^	0
48	1.6 × 10^9^	97.8	9.3 × 10^6^	0	1.1 × 10^7^	0	1.5 × 10^7^	0
60	1.4 × 10^9^	100.0	8.5 × 10^5^	0	8.7 × 10^5^	0	8.1 × 10^5^	0

After growth in LB medium for 60 h, the viability of the strains was reflected in the cell growth curves (Fig. 3d), and the number of viable bacteria and sporulation frequency were quantified (Table 2). There were some differences between the parent strain and the mutants, whereby the OD_600_ of the control was stabilized at about 2.0, while that of the *sig*E and *sig*F mutants gradually decreased as shown in Fig. 3d, which may be attributed to the insufficiently rich medium and not to cell lysis as shown in Fig. 3a3. When the two strains cultured in BHI medium, the cell population remained steady at about 2.5 × 10^9^ cfu/mL (data not shown) throughout the incubation period of 72 h, which was in accordance with the data for same strains cultured in LB medium for 36 h (Table 2). However, although the *spo*0A mutant did not present any obvious defects in cell growth from the exponential to the early stationary phase (Fig. 3d), its cell density and viable cell count markedly decreased after 24 h (Table 2). Together with the changes cellular morphology observed under the optical microscope (Fig. 3a2) we concluded that autolysis likely occurred in the *spo*0A mutant. Therefore, it can be said that despite the changes of sporogenicity, there was no indication of loss of viability or cell lysis in the *sig*E and *sig*F mutants when grown in LB medium and the *spo*0A mutant was prone to cell lysis.

### Production of alkaline protease by the different spore-formation mutants

**Fig. 4:**
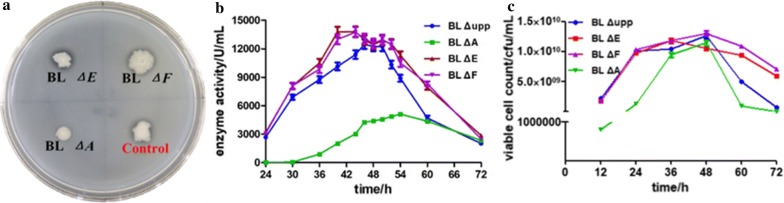
Alkaline protease synthesis measured in different strains. **a** Detection of protease in Buttermilk plate; **b** alkaline protease enzyme activity assay of the mutants and wild-type strain; **c** the viable cell count of the mutants and wild-type strain

The results of the initial buttermilk plate tests suggested that the *sig*E and *sig*F mutant strains differed slightly from the control strain in the amounts of extracellular protease they produced. However, the *spo*0A deletion resulted in a loss of the ability to synthesize protease as evidenced by the smaller hydrolysis zones, which indicated that the synthesis of protease was seriously affected by the DNA binding protein Spo0A (Fig. 4a). We next monitored the synthesis of alkaline protease when the strains were grown in fermentation medium for 72 h (Fig. 4b). The protease yield was higher in the *sig*E and *sig*F mutants than in the parent strain, particularly later in the growth cycle, whereby the enzyme activity of the two mutants decreased more slowly. The enzyme yield peaked after incubation for about 40 h in both mutants and then remained steady for about 12 h, while the parent strain reached its maximum enzyme activity at 46 h, and the stationary phase lasted only about 6 h. By contrast, the synthesis of protease greatly repressed in the *spo*0A mutant, which reached the highest enzyme activity of only 5100 U/mL after incubation for 54 h.

However, considering the possible effect of biomass on the synthesis and accumulation of the enzyme, a viable count was performed at different culture times to assess alkaline protease activity per unit biomass (Fig. 4c). We found that, the cell growth was seriously influenced by the Spo0A in the fermentation medium, but there was no significant difference between the other two mutants and the wild-type strain except that the viable cell count of the two mutants was higher than that of the control strain in the later growth stage. Thus, the *sig*E and *sig*F mutants also performed somewhat better than the parent, but the deletion of *spo*0A resulted in a loss of the ability to synthesize extracellular protease. All the mutants remained asporous throughout the whole fermentation process and the morphological changes were similar to those observed when the strains were cultured in LB medium. These results indicated that the sporulation-deficiency of *sig*E and *sig*F had no negative effect on protease synthesis, and that BL *ΔE* and BL *ΔF* may be excellent alternative strains for industrial enzyme production.

### Transcriptional analysis of the alkaline protease gene in the mutant strains

**Fig. 5:**
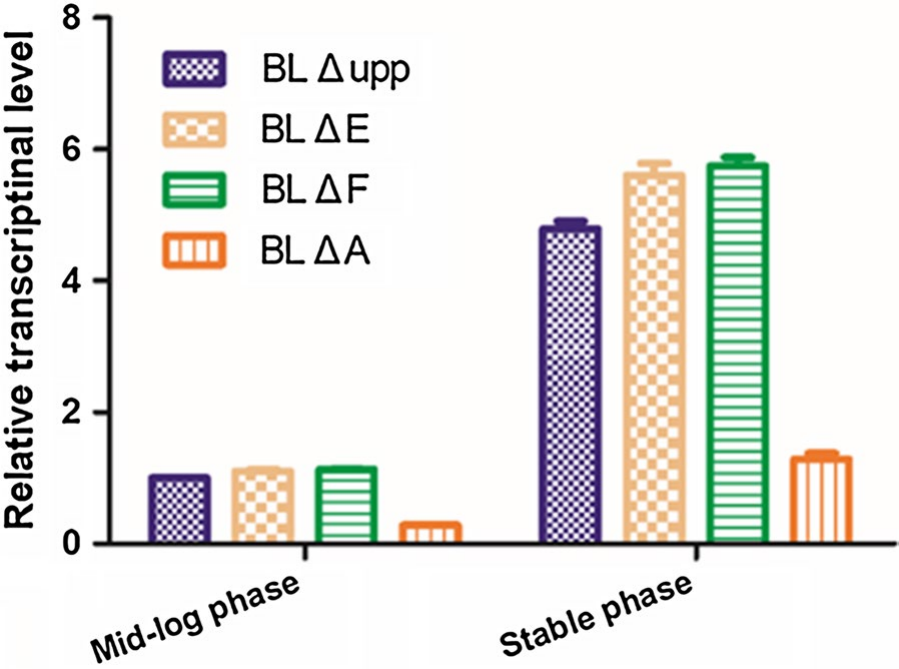
The relative gene expression levels of *apr*E at the mid-log phase and early stable phase

In view of the dramatic difference between the *spo*0A mutant and the other three strains in alkaline protease activity when cultivated in fermentation medium, the relative gene expression levels of the *aprE* gene were evaluated at the mid-log phase (26 h) and the early stable phase (40 h) in the parental strain, BL *ΔE* and BL *ΔF* during the protease production process, as well as at 40 h and 48 h, respectively in BL *ΔA*. As shown in Fig. 5, the *apr*E transcriptional level in BL *ΔE* and BL *ΔF* were increased by about 25% compared to the control strain at both stages, while in the BL *ΔA* it was dramatically affected, reaching only about 0.3-fold of the control value. Importantly, in the early stable phase the *apr*E transcriptional levels of all the strains were increased about fourfold compared with the mid-log phase, indicating that *apr*E expression was initiated in the late phase of cell growth.

### Enhanced protease production in a 5-L fermenter

**Fig. 6:**
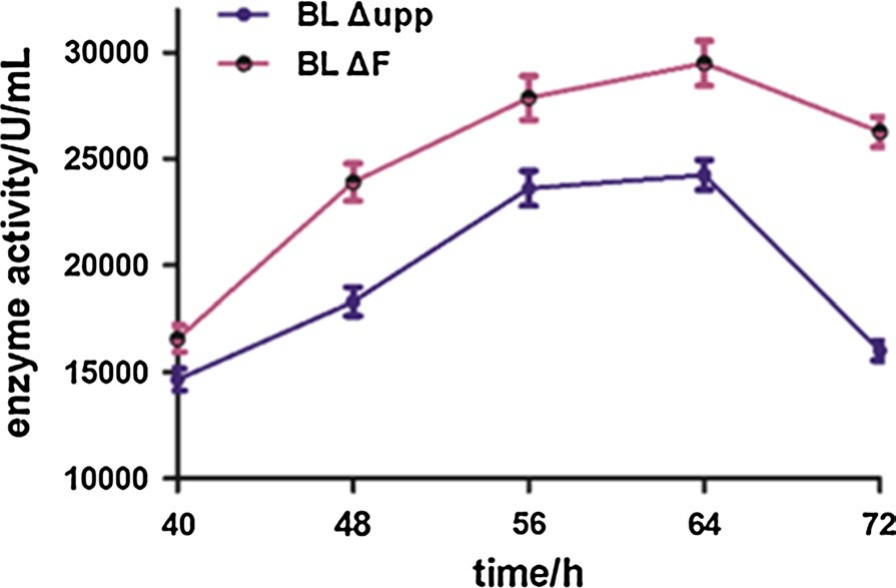
Production of alkaline protease in 5 L fermenter

Fed-batch fermentation was applied to improve the yield of alkaline protease using BL *ΔF* and the control strain based on the studies of metabolic processes during batch fermentation in a 5-L auto-fermenter. The concentration of biomass (as a proxy for the viable bacteria count) and reducing sugars were determined in real time to control the pH, dissolved oxygen and to optimize the fed-batch fermentation strategy by making fine adjustments. The changes of different parameters during the fermentation were shown in Table 3. The pH, concentration of reducing sugars and dissolved oxygen was as far as possible kept at about 7.3, 15% and 35%, respectively, meanwhile the highest relative activity of alkaline protease was obtained. As shown in Fig. 6, the production of alkaline protease remained at a relatively higher level of 19.7% in BL *ΔF* compared with that obtained in the control strain BL *Δupp*. Also, when the incubation time was 48 h, the enzyme activity of BL *ΔF* reached to 23,910 ± 885 U/mL, and up to 29,494 ± 1053 U/mL at 64 h, showing that the stable period was longer by about 16 h in the 5-L fermenter. It is worth mentioning that the enzyme yield of BL *ΔF* declined more slowly, and the result resembled that obtained in the shake flasks, which might be due to the change trend of biomass (Table 3).

**Table 3:** Changes of different parameters during fermentation in 5 L fermenter of BL *Δupp* and BL *ΔF*

Time/h	BL *Δupp*				BL *ΔF*			
	VC/cfu/mL	DO_2_/%	pH	RS/%	VC/cfu/mL	DO_2_/%	pH	RS/%
0	5.71 × 10^4^	90	7.28	23.67	6.33 × 10^4^	90	7.26	24.18
12	4.95 × 10^8^	53.4	7.13	19.70	5.86 × 10^8^	50.7	7.16	20.03
24	1.23 × 10^10^	32.0	6.97	16.76	1.44 × 10^10^	33.6	7.23	16.85
36	1.87 × 10^10^	37.9	7.11	16.64	2.09 × 10^10^	34.9	7.19	16.54
48	3.62 × 10^10^	35.7	7.10	15.89	3.13 × 10^10^	36.3	7.15	16.16
60	3.47 × 10^10^	36.1	7.17	16.12	2.95 × 10^10^	35.5	7.19	17.04
72	1.96 × 10^10^	37.4	7.09	16.06	2.46 × 10^10^	36.2	7.11	16.51

*VC* viable counts, *DO*_*2*_ dissolved oxygen, *RS* represents reducing sugar

## Discussion

Gene excision and integration is a frequently-used procedure for engineering specific mutations in *Bacillus* and other Gram-positive bacteria [33–35]. Over the past several years, the CRISPR-Cas-based adaptive immune system from bacteria and archaea has been repurposed for versatile genome editing or transcriptional regulation in many different species. In particular, methods based on the Type II CRISPR/Cas9 system have been widely used in *Bacillus* [21, 36], and have also been harnessed for genome editing to construct targeted mutants in *B. licheniformis* by our own group [7]. In this work, we further demonstrated that the procedure could be applied to achieve efficient chromosome editing to successfully construct three sporulation-deficient strains.

*Bacillus licheniformis* has the generally recognized as safe (GRAS) status, which has promoted its development as a host strain in the pharmaceutical and nutraceutical industries [37]. However, endospore formation is a harmful undomesticated characteristic that is activated in response to an adverse growth environment and can strongly impact the production safety [14]. Several general strategies for the successful development of industrial hosts were introduced to obtain *Bacillus* cellular factories with improved cellular performances [38]. Given this, we realized that deleting sporulation-related genes might lead to an improvement of enzyme productivity. Our understanding of the genetics of sporulation in *B. licheniformis* is now at an advanced stage, and with the aid of the complete genome sequence, spore-related genes have been well characterized. To initiate sporulation, the master regulator of sporulation Spo0A activates the expression of the sporulation-specific RNA polymerase σ factor, SigF, in the forespore. This sigma factor is the product of the *spoII*AC gene and the first sigma factor in the regulatory cascade that controls sporulation [39]. SigF in turn activates SigE protein, the first mother-cell-specific sigma factor [26]. Thus, in this case, we constructed *spo*0A, *sig*E and *sig*F mutants to investigate the effects of sporulation on protease production.

As the results shown in Fig. 3a3 indicate, normal asymmetric septum formation was inhibited in both *sig*E-deleted and *sig*F-defective mutants, which displayed ‘bipolar’ or ‘disporic’ cells with the septum formed at both ends, which resulted in mature endospore formation being blocked as reported previously [20, 40]. Because they are sporulation-specific factors that are active during sporulation stage II, depletion of the two sigma factors had no effect on cell growth and bacteriolysis. However, deletion of the DNA-binding protein Spo0A had obvious detrimental effects on cell growth, such as changes cellular and colony morphology, increased cell lysis, and reduction of the enzyme yield, which can be explained by the “transition state” (such as motility, protease production, competence and biofilm formation) resulting from the initial activation of Spo0A [18]. Some researchers found that mutations affecting stage II of sporulation have little effect on the production of extracellular enzymes because they are generally synthesized in the late exponential phase and the early stationary phase [41]. In fact, such changes could even improve cellulase productivity [22], whereas protease activity was significantly decreased in the *spo*0A mutant [23]. Fortunately, when we investigated the effect of sporulation on alkaline protease synthesis, a yield-enhanced sporulation-deficient *B. licheniformis* called BL *ΔF* was successfully established, whose *apr*E gene transcriptional level was apparently improved. Importantly, the BL *ΔF* was superior in operation simpleness, energy conservation, and target product controlling effect due to the tremendously prolonged stable phase of enzyme production; furthermore, the enzyme yield declined more slowly compared with the control strain, which has great value for industrial applications [15]. The advanced performance could help avoid large losses of the target enzyme during industrial production [42]. The BL *ΔF* strain was completely asporogenic, absolutely stable and unable to revert to sporogeny, while also outperforming the parent strain in the synthesis and secretion of extracellular enzymes.

Traits favorable for the synthesis of extracellular enzymes are induced during the transition from exponential growth to the stationary phase in batch culture, suggesting that sporulation and protease production might be coregulated [43]. However, whether the metabolic activities of the cell led to the improvement of protease activity remains unclear. Furthermore, the link between Spo0A and the production of extracellular enzymes in *B. licheniformis* was not systematically investigated. We will next study the genes related to the transcription, translation and secretion apparatuses, as well as cell cycle-related, ATP-synthesis-related, transporter-related and stress response-related genes via RNA sequencing, genome and transcriptome analyses of the mutants and control strain. Although its capacity to produce alkaline protease was severely reduced, the *spo*0A mutant might be a better host for the production of extracellular proteins, because its function resembles the multiple-protease-deficient mutants with very low protease activity. If the role of Spo0A in alkaline protease synthesis can be confirmed in future studies, the mutants can be employed directly to produce other target proteins with higher efficiency. Thus, we will further investigate the regulatory mechanisms of Spo0A protein in *B. licheniformis* in the future.

## Conclusions

The major aim of this work was to investigate the effect of sporulation on protease production and engineered an industrial host with excellent performance. We constructed three asporogenous strains of *B. licheniformis* using a previously established CRISPR/Cas9 system. All the mutants were asporogenic as predicted, and the mutations affecting stage II of sporulation increased the production of alkaline protease efficiently. Therefore, we concluded that introducing a deletion in the *sig*E or *spo*IIAC gene can produce a mutant that retains the important industrial properties and preferable characteristics, while prolonging the stable phase of enzyme activity and leading to a slower decline, which has great application value in industrial production. In summary, this work provides an industrial strain with excellent performance and a novel strategy to enhance protease synthesis.

## Additional file

**Additional file 1: Table S1.** Main oligonucleotides used in this study. **Table S2.** Main oligonucleotides used in backcrossed experiment. **Fig. S1.** Confirmation of complementation of *sig*F, *spo*0A and *sig*E. Agarose gel electrophoresis: *spo*IIAC, insertion 756bp; *spo*0A, insertion 459bp; sigE insertion 612bp. M: nucleic acid marker; NC: The negative control was obtained by colony PCR of the specific sporulation-deficient mutant.

## Data Availability

All data generated or analyzed during this study are included in this published article.

## References

[1] Ibrahim AS, Al-Salamah AA, El-Badawi YB, El-Tayeb MA, Antranikian G (2015). Detergent-, solvent- and salt-compatible thermoactive alkaline serine protease from halotolerant alkaliphilic *Bacillus* sp. NPST-AK15: purification and characterization. Extremophiles.

[2] Yildirim V, Baltaci MO, Ozgencli I, Sisecioglu M, Adiguzel A, Adiguzel G (2017). Purification and biochemical characterization of a novel thermostable serine alkaline protease from *Aeribacillus pallidus* C10: a potential additive for detergents. J Enzyme Inhib Med Chem.

[3] Abdel-Naby MA, Ahmed SA, Wehaidy HR, El-Mahdy SA (2017). Catalytic, kinetic and thermodynamic properties of stabilized *Bacillus stearothermophilus* alkaline protease. Int J Biol Macromol.

[4] Hammami A, Hamdi M, Abdelhedi O, Jridi M, Nasri M, Bayoudh A (2017). Surfactant- and oxidant-stable alkaline proteases from *Bacillus invictae*: characterization and potential applications in chitin extraction and as a detergent additive. Int J Biol Macromol.

[5] Schallmey M, Singh A, Ward OP (2004). Developments in the use of *Bacillus* species for industrial production. Can J Microbiol.

[6] Wiegand S, Voigt B, Albrecht D, Bongaerts J, Evers S, Hecker M, Daniel R, Liesegang H (2013). Fermentation stage-dependent adaptations of *Bacillus licheniformis* during enzyme production. Microb Cell Fact.

[7] Zhou C, Liu H, Yuan F, Chai H, Wang H, Liu F, Li Y, Zhang H, Lu F (2019). Development and application of a CRISPR/Cas9 system for *Bacillus licheniformis* genome editing. Int J Biol Macromol.

[8] Wei X, Zhou Y, Chen J, Cai D, Wang D, Qi G, Chen S (2015). Efficient expression of nattokinase in *Bacillus licheniformis*: host strain construction and signal peptide optimization. J Ind Microbiol Biotechnol.

[9] Harwood CR, Mouillon JM, Pohl S, Arnau J (2018). Secondary metabolite production and the safety of industrially important members of the *Bacillus subtilis* group. FEMS Microbiol Rev.

[10] Voigt B, Schroeter R, Schweder T, Jurgen B, Albrecht D, van Dijl JM, Maurer KH, Hecker M (2014). A proteomic view of cell physiology of the industrial workhorse *Bacillus licheniformis*. J Biotechnol.

[11] Grimbergen AJ, Siebring J, Solopova A, Kuipers OP (2015). Microbial bet-hedging: the power of being different. Curr Opin Microbiol.

[12] Hecker M, Volker U (2004). Towards a comprehensive understanding of *Bacillus subtilis* cell physiology by physiological proteomics. Proteomics.

[13] Dijl J, Hecker M (2013). *Bacillus subtilis*: from soil bacterium to supersecreting cell factory. Microb Cell Fact.

[14] Bressuire-Isoard C, Broussolle V, Carlin F (2018). Sporulation environment influences spore properties in *Bacillus*: evidence and insights on underlying molecular and physiological mechanisms. FEMS Microbiol Rev.

[15] Li T, Chen XB, Chen JC, Wu Q, Chen GQ (2014). Open and continuous fermentation: products, conditions and bioprocess economy. Biotechnol J.

[16] Tocheva EI, Ortega DR, Jensen GJ (2016). Sporulation, bacterial cell envelopes and the origin of life. Nat Rev Microbiol.

[17] Keynan A, Sandier N (1984). Spore research in historical perspective. The bacterial spore.

[18] Hilbert DW, Piggot PJ (2004). Compartmentalization of gene expression during *Bacillus subtilis* spore formation. Microbiol Mol Biol Rev.

[19] Zhang J, Zong W, Hong W, Zhang ZT, Wang Y (2018). Exploiting endogenous CRISPR-Cas system for multiplex genome editing in *Clostridium tyrobutyricum* and engineer the strain for high-level butanol production. Metab Eng.

[20] Mascher G, Mertaoja A, Korkeala H, Lindstrom M (2017). Neurotoxin synthesis is positively regulated by the sporulation transcription factor Spo0A in *Clostridium botulinum* type E. Environ Microbiol.

[21] Zhang K, Duan X, Wu J (2016). Multigene disruption in undomesticated *Bacillus subtilis* ATCC 6051a using the CRISPR/Cas9 system. Sci Rep.

[22] Ara K, Ozaki K, Nakamura K, Yamane K, Sekiguchi J, Ogasawara N (2007). *Bacillus* minimum genome factory: effective utilization of microbial genome information. Biotechnol Appl Biochem.

[23] Eugenio F, Sandra M, James A (1986). Effect of stage 0 sporulation mutations on subtilisin expression. J Bacteriol.

[24] Kodama T, Endo K, Ara K, Ozaki K, Kakeshita H, Yamane K, Sekiguchi J (2007). Effect of *Bacillus subtilis spo*0A mutation on cell wall lytic enzymes and extracellular proteases, and prevention of cell lysis. J Biosci Bioeng.

[25] Abraham L (2000). Control of sporulation initiation in *Bacillus subtilis*. Curr Opin Microbiol.

[26] Piggot PJ, Hilbert DW (2004). Sporulation of *Bacillus subtilis*. Curr Opin Microbiol.

[27] Lapidus A, Galleron N, Andersen J, Ehrlich S, Sorokin A (2002). Co-linear sca¡old of the *Bacillus licheniformis* and *Bacillus subtilis* genomes and its use to compare their competence genes. FEMS Microbiol Lett.

[28] Fleming AB, Tangney M, Jorgensen PL, Diderichsen B, Priest FG (1995). Extracellular enzyme synthesis in a sporulation-deficient strain of *Bacillus licheniformis*. Appl Environ Microbiol.

[29] Zhang G, Wang W, Deng A, Sun Z, Zhang Y, Liang Y, Che Y, Wen T (2012). A mimicking-of-DNA-methylation-patterns pipeline for overcoming the restriction barrier of bacteria. PLoS Genet.

[30] Ong KS, Aw YK, Lee LH, Yule CM, Cheow YL, Lee SM (2016). *Burkholderia paludis* sp. nov., an antibiotic-siderophore producing novel *Burkholderia cepacia* complex species, isolated from Malaysian Tropical peat swamp soil. Front Microbiol.

[31] National food safety standard of the People’s Republic of China: The National Standardization Administration Commission GB/T 4789.35-2010, Food microbiological examination: Lactic acid bacteria. 2010.

[32] State Administration for Quality Supervision and Inspection and Quarantine of the People’s Republic of China: The National Standardization Administration Commission GB/T 23527-2009, Proteinase preparations. 2009.

[33] Indranil B, Alexandra G, Ehrlich S, Emmanuelle M (1993). High-efficiency gene inactivation and replacement system for gram-positive bacteria. J Bacteriol.

[34] Martin T, Per L, Berge D, Steen T (1995). A new method for integration and stable DNA amplification in poorly transformable bacilli. FEMS Microbiol Lett.

[35] Ireton K, Rudner DZ, Siranosian KJ, Grossman AD (1993). Integration of multiple developmental signals in *Bacillus subtilis* through the Spo0A transcription factor. Genes Dev.

[36] Li K, Cai D, Wang Z, He Z, Chen S (2018). Development of an efficient genome editing tool in *Bacillus licheniformis* using CRISPR-Cas9 Nickase. Appl Environ Microbiol.

[37] Jin P, Zhang L, Yuan P, Kang Z, Du G, Chen J (2016). Efficient biosynthesis of polysaccharides chondroitin and heparosan by metabolically engineered *Bacillus subtilis*. Carbohydr Polym.

[38] Dong H, Zhang D (2014). Current development in genetic engineering strategies of *Bacillus* species. Microb Cell Fact.

[39] Wu J, Howard M, Piggot P (1989). Regulation of transcription of the *Bacillus subtilis spo*IIA locus. J Bacteriol.

[40] Al-Hinai MA, Jones SW, Papoutsakis ET (2015). The *Clostridium* sporulation programs: diversity and preservation of endospore differentiation. Microbiol Mol Biol Rev.

[41] Priest FG (1977). Extracellular enzyme synthesis in the genus *Bacillus*. Bacteriol Rev.

[42] Gupta R, Beg QK, Lorenz P (2002). Bacterial alkaline proteases: molecular approaches and industrial applications. Appl Microbiol Biotechnol.

[43] Kirk DG, Zhang Z, Korkeala H, Lindstrom M (2014). Alternative sigma factors SigF, SigE, and SigG are essential for sporulation in *Clostridium botulinum* ATCC 3502. Appl Environ Microbiol.

