# Supplementary material for: Optimization of alkaline protease production by rational deletion of sporulation related genes in Bacillus licheniformis
Source: Microb Cell Fact. 2019 Jul 25;18:127. doi: 10.1186/s12934-019-1174-1 (PMC6657089; doi:10.1186/s12934-019-1174-1)
Supplement: Supplementary file 1 — Additional file 1: Table S1. Main oligonucleotides used in this study. Table S2. Main oligonucleotides used in backcrossed experiment. Fig. S1. Confirmation of complementation of sigF, spo0A and sigE. Agarose gel electrophoresis: spoIIAC, insertion 756bp; spo0A, insertion 459bp; sigE insertion 612bp. M: nucleic acid marker; NC: The negative control was obtained by colony PCR of the specific sporulation-deficient mutant. [file 12934_2019_1174_MOESM1_ESM.docx]

Table S1 Main oligonucleotides used in this study

| Primer name | Sequence (5'–3') | Purpose |
| --- | --- | --- |
| F-LF | GACAAATGGTCCAAACTAGTGGTTCACCTTGCCCGTCACC | *Spe* I*/Spe* I  PCR of homologous template for deletion of *sigF* gene, gene deletion |
| F-LR | GGAGATTTGTTGGCTGCTCATGTTGCAGGCA |  |
| F-RF | ATGAGCAGCCAACAAATCTCCCTTAATTGCAAAGCG |  |
| F-RR | CAATAATGCTGAGCTCACTAGTGTGCTCGGCAGATATAAGGAGATTAAG |  |
| 0A-LF | GACAAATGGTCCAAACTAGTACATGCAGGTATGTCCAAAAAGTG | *Spe* I*/Spe* I  PCR of homologous template for deletion of *spo0A* gene, gene deletion |
| 0A-LR | CGACGTGCTTACTGCAAGCCGCGTCGAA |  |
| 0A-RF | GGCTTGCAGTAAGCACGTCGGGCTCCTTG |  |
| 0A-RR | CAATAATGCTGAGCTCACTAGTGAATGGATGCTGTCTGAAGCG |  |
| E-LF | GACAAATGGTCCAAACTAGTGATATCCCTGAGTGACCGTGAG | *Spe* I*/Spe* I  PCR of homologous template for deletion of *sigE* gene |
| E-LR | AAGCGACGAAAGGCTCCGAAAAGAATTCAAC |  |
| E-RF | TTCGGAGCCTTTCGTCGCTTTTCAGCCC |  |
| E-RR | CAATAATGCTGAGCTCACTAGTCTTCATTTCACCGGATTGATTG |  |
| 0A-VF | CCAACAGCCAGAGTCGTACCAG | diagnostic PCR of the mutation and sequence-spo0A |
| 0A-VR | TCGTCAATGATCCGACGAGC |  |
| F-VF | GACCGATGCTGTACGGAACCTG | diagnostic PCR of the mutation and sequence-*sigF* |
| F-VR | CCGACATTCGCCATATTGTCATG |  |
| E-VF | CGATCTCCTGAGCAGTCGGC | diagnostic PCR of the mutation and sequence-*sigE* |
| E-VR | CAATACGAAGAACGCGTCCG |  |
| 16S-F | CGTTGCTCCGTCAGACTTTC | fluorogenic quantitative PCR of 16s rRNA |
| 16S-R | GGGCTAATACCGGATGCTTG |  |
| AP-F | CGGATCTTCAGGAAACACG | fluorogenic quantitative PCR of *apr*E |
| AP-R | ATGAGGAGAAGCCATTGACG |  |

Table S2 Main oligonucleotides used in backcrossed experiment

| Primer name | Sequence (5'–3') | Purpose |
| --- | --- | --- |
| F-CLF | **same as F-LF** | *Spe* I*/Spe* I  PCR of donor template for complementation of *sigF* gene |
| F-CLR | TGAAAGCTGAGGCTGCTCATGTTGCAGGCA |  |
| F-CF | ATGAGCAGCCTCAGCTTTCAAAATGGTCCATCTG |  |
| F-CR | GAGATTTGTTATGGATGTGGAGGTTAAAAAAGAAAAC |  |
| F-CRF | CCACATCCATAACAAATCTCCCTTAATTGCAAAGCG |  |
| F-CRR | **same as F-RR** |  |
| 0A-CLF | **same as 0A-LF** | PCR of donor template for complementation of *spo0A* gene |
| 0A-CLR | ATTCAACACGACTGCAAGCCGCGTCGAA |  |
| 0A-CF | GGCTTGCAGTCGTGTTGAATTTTTTGGCGATATC |  |
| 0A-CR | CGACGTGCTTGTGCTGGATATAATCATGCCTCATC |  |
| 0A-CRF | GGCTTGCAGTAAGCACGTCGGGCTCCTTG |  |
| 0A-CRR | **same as 0A-RR** |  |
| E-CLF | **same as E-LF** | PCR of donor template for complementation of *sigE* gene |
| E-CLR | GATGGTTTAAAGGCTCCGAAAAGAATTCAAC |  |
| E-CF | TTCGGAGCCTTTAAACCATCTTGTTGAATTCTTTTCG |  |
| E-CR | AAGCGACGAAATTTATTATATCGGCGGAAGCGAG |  |
| E-CRF | TTCGGAGCCTTTCGTCGCTTTTCAGCCC |  |
| E-CRR | **same as E-RR** |  |
| 0A-CVF | **same as 0A-VF** | diagnostic PCR of the mutation |
| 0A-CVR | **same as 0A-VR** |  |
| F-CVF | **same as F-VF** | diagnostic PCR of the mutation |
| F-CVR | **same as F-VR** |  |
| E-CVF | **same as E-VF** | diagnostic PCR of the mutation |
| E-CVR | **same as E-VR** |  |


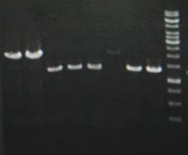


**NC**


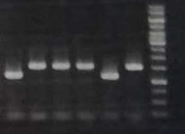


**NC**

***sig*F**

***spo*0A**

***sig*E**


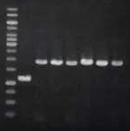


**NC**

**1000**

**2000**

**2000**

**1000**

**1000**

**1500**

***sig*E**

**M**

**M**

**M**

Fig. S1 Confirmation of complementation of *sig*F, *spo*0A and *sig*E

Agarose gel electrophoresis: *spo*IIAC, insertion 756bp; *spo*0A, insertion 459bp; *sig*E insertion 612bp. M: nucleic acid marker; NC: The negative control was obtained by colony PCR of the specific sporulation-deficient mutant.
